# Central Adiposity Versus Body Mass Index in Assessment of Adiposity-Related Differences in Physical Activity Among Schoolchildren Aged 5–12 Years in High-Risk Neighborhoods

**DOI:** 10.3390/sports14090394

**Published:** 2026-09-07

**Authors:** Matthew J. Barenie, Michael R. Thomsen, Harneet Kaur, Erin K. Howie, Christopher M. Murphy, Kari A. Weber

**Affiliations:** 1 Center for the Study of Obesity, Fay W. Boozman College of Public Health, University of Arkansas for Medical Sciences, 4301 W. Markham, Slot 820, Little Rock, AR 72205, USA; 2 Department of Health, Kinesiology, and Sport, College of Education and Professional Studies, University of South Alabama, 171 Student Services Drive, Mobile, AL 36688, USA; 3 Department of Health, Human Performance and Recreation, University of Arkansas, 155 Stadium Drive, Fayetteville, AR 72701, USA; 4 Department of Epidemiology, Fay W. Boozman College of Public Health, University of Arkansas for Medical Sciences, 4301 W. Markham, Slot 82, Little Rock, AR 72205, USA

**Keywords:** accelerometry, central adiposity, obesity surveillance, waist circumference, waist-to-height ratio

## Abstract

**Purpose:** Body mass index (BMI) is widely used in childhood obesity surveillance despite limitations in capturing central adiposity, which has been hypothesized to relate to reduced participation in movement. This study compared associations between z-score waist circumference (zWC), z-score waist-to-height ratio (zWHR), and BMI and device-measured physical activity in predominantly African American, low-income schoolchildren. **Methods:** This cross-sectional study included 264 schoolchildren aged 5–12 years (146 girls, 118 boys; mean age 8.5 ± 1.8 years; ~86% African American; ~93% eligible for free/reduced-price lunch). Anthropometrics and free-living PA (Actigraph GT9X) were measured. Regression models evaluated associations between each anthropometric z-score and PA intensities, adjusting for age, sex, wear time, and school. **Results:** Measures of central adiposity showed inverse associations with physical activity that were of similar or slightly greater magnitude than those for BMI, although differences between models were modest. All three adiposity measures (zWC, zWHR, zBMI) were significantly and negatively associated with total moderate–vigorous physical activity (MVPA), only in girls. A one-unit increase in zWC was associated with a decrease of 3.334 min/day in MVPA. The largest negative associations were observed for zWC and zWHR with vigorous activity. **Conclusions:** In this population of schoolchildren, waist-based measures showed inverse associations with physical activity of similar or slightly greater magnitude than BMI, particularly among girls. Although modest differences in model fit prohibit claiming superiority over BMI, these findings support utilizing waist-based measures as practical, low-cost complements to traditional BMI in school-based surveillance and activity targeting.

## 1. Introduction

Visceral adiposity, characterized by an increased amount of adipose tissue surrounding intra-abdominal organs, is linked to many adverse health outcomes, including elevated cardiometabolic risk [1], asthma [2], non-alcoholic fatty liver disease risk [3], and lower cognitive function [4] in children. Strategies to reduce visceral adiposity are crucial, as it is associated with low-grade inflammation [5], elevated blood pressure [6], and insulin resistance [7]. Widely used measures of total adiposity, such as body mass index, may significantly underestimate body fat in children [8,9] and do not measure regional adiposity, such as visceral adiposity; therefore, alternative measures that better reflect visceral fat are needed. The waist-to-height ratio (WHR) is a simple, low-cost, noninvasive metric that may better predict central adiposity than BMI or body weight [10,11]. The clinical relevance of waist circumference in pediatric populations has been recognized for over a decade, demonstrating its utility as a practical indicator of adiposity and cardiometabolic risk in children [12]. Studies recommend that the WHR measure is necessary in routine health screenings of children [13] and is also recommended as a home-based screening tool for obesity [14]. However, not all research finds that WHR is superior to body mass index for associations with health outcomes, as some evidence suggests that body mass index and WHR predict cardiometabolic risk similarly [15,16]. In summary, the evidence is mixed: several studies report advantages of waist-based measures in detecting cardiometabolic risk, while others find performance comparable to BMI. The relative utility of each measure appears to depend on the outcome examined, the population studied, and the reference values used. The present study is therefore framed as a comparison of the associations each measure shows with physical activity in one specific population, rather than as a test of the general superiority of one measure over another.

Various optimal health WHR cutoffs exist: 0.50 [17,18,19] and 0.49 [20] are widely used; however, to better optimize cardiometabolic risk detection, there is evidence that optimal thresholds vary by ethnicity, sex, and age [21]. A recent systematic review highlights waist circumference, often expressed as age- and sex-standardized z-scores, as a meaningful measure in school-based obesity prevention interventions, given its sensitivity to cardiometabolic risk and responsiveness to behavior change [22]. As a result, central adiposity measures have been increasingly recommended as feasible, low-cost tools for pediatric surveillance and intervention research, alongside or in place of BMI.

Physical activity serves as a critical “test case” for evaluating these metrics because it is a primary behavioral determinant of metabolic health and a key target for obesity surveillance [23]. While traditional models often emphasize physical activity as a means to reduce adiposity, emerging evidence suggests a bidirectional relationship in which excess central adiposity and reduced participation in higher-intensity movement may reinforce one another over time [24]. Whether central adiposity constrains movement, whether lower activity contributes to adiposity accumulation, or whether both operate simultaneously cannot be determined from cross-sectional data. If central adiposity is more strongly associated with these activity limitations than BMI, relying solely on BMI for school-based screenings may result in a “sensitivity gap,” failing to identify children at the highest risk of physical inactivity and its subsequent cardiometabolic consequences [10]. Racial disparities in childhood obesity and ensuing cardiometabolic effects remain a public health challenge, as African American children often experience higher rates of severe obesity compared to their white peers [25]. Identifying which measures of adiposity most consistently track physical inactivity in this high-risk population may help ensure that screening reaches those most vulnerable.

Relatively few studies have compared associations between multiple anthropometric exposures, z-score waist circumference (zWC), z-score waist-to-height ratio (zWHR), and BMI, and device-measured physical activity outcomes in predominantly African American, low-income schoolchildren at high risk for cardiometabolic outcomes [26]. Evidence suggests the WHR tracks more closely with adiposity and its association with vigorous exercise than the commonly used body mass index [27]. Instead of vigorous exercise, other research groups recommend examining total daily light physical activity and weight status in children using accelerometry [28]. Therefore, more data is needed to elucidate the role of central adiposity measures and device-measured physical activity intensities in schoolchildren. Furthermore, young individuals from low-income backgrounds experience less sleep, engage in more recreational screentime, and have fewer opportunities for leisure-time physical activity than their higher-income counterparts [29,30,31]. Hence, the purpose of this work is to compare associations between multiple anthropometric variables, particularly central adiposity measures and device-measured physical activity intensities, among predominantly African American, low-income schoolchildren. We hypothesized that zWC and zWHR would show stronger and more consistent inverse associations with device-measured MVPA and vigorous physical activity than zBMI, and that these associations would be most evident at higher activity intensities.

## 2. Materials & Methods

### 2.1. Participants

This study included 264 unique schoolchildren across five academic semesters (Spring 2023, Fall 2023, Spring 2024, Fall 2024, and Spring 2025) from four elementary schools located in an urban southern metropolitan area of the United States. To ensure independent observations, a child’s data was used only once, and the longest valid accelerometer wear time of the possible five semesters was selected. This work is part of a larger study examining the impact of green schoolyards on schoolchildren’s physical activity [26,32,33]. The school’s enrollment was ~86% African American. In total, ~93% of students were eligible for free and reduced-price lunch [32] at the time of data collection. Parental consent and then child assent were obtained at the school. Ethical approval for this study was received by the University of Arkansas for Medical Sciences Institutional Review Board (Protocol #274741) and the participating school district.

### 2.2. Anthropometry

Children’s anthropometric measures, including height, weight, and waist circumference, were measured by trained research assistants. Standardized protocols with a free-standing portable height rod (0044-0392-0M, Detecto, Webb City, MO, USA) and an electronic scale (BF-689, Tanita, Arlington Heights, IL, USA) for body weight were followed. The waist circumference at the umbilicus was measured twice with a tape measure (BalanceFrom Body Tape Measure, Ontario, CA, USA) and averaged for analysis [34]. BMI was calculated from measured height and weight and expressed as a z-score using CDC data [35]. Sharma and colleagues provided the LMS values needed for age- and sex-specific WC and WHR z-scores [36]. Anthropometric exposures were expressed as z-scores rather than absolute values for two reasons. First, the sample spans a period of substantial growth, across which absolute waist circumference and waist-to-height ratio vary markedly with age and differ systematically by sex independently of adiposity; absolute values would therefore confound adiposity with normal growth. Second, standardization places all three exposures in a common metric, allowing their coefficients to be compared directly, which is central to this study’s aim.

### 2.3. Physical Activity Measurement via Accelerometry

Free-living physical activity was measured using waist-worn Actigraph GT9X accelerometers (ActiGraph, Pensacola, FL, USA). Schoolchildren were asked to wear the devices on the right hip for seven consecutive days, 24 h per day, except during water-based activities. Additionally, parents were asked to report in- and out-of-bed times and when the monitor was removed via a sleep diary, in accordance with existing protocols [37,38,39,40,41]. The study staff instructed the children on how to wear the device and answered any questions they had. Data were processed using ActiLife software (ActiGraph, Pensacola, FL, USA, version 6.13.4). Thirty-six percent of parents completed sleep diaries. Two coders independently identified rest intervals to estimate waking wear time from in-bed and out-of-bed times, using visual inspection of the data and incorporating diary times when available [42]. A third coder re-examined the data for times that differed by more than 15 min. Non-wear time was calculated using the Troiano algorithm with a 60-s minimum wear period [43]. At least 10 h of waking wear time per day was required, along with a total of three days [44,45,46]. A threshold daily average of 60 min of moderate-to-vigorous physical activity (MVPA) was used to indicate whether subjects met current physical activity guidelines [47]. The primary physical activity variable was the number of minutes in MVPA.

### 2.4. Statistical Analyses

Descriptive statistics, including means, standard deviations, and frequencies, were calculated to characterize the overall sample (*N* = 264) and were stratified by sex. Multiple linear regression models were used to identify factors associated with MVPA. Separate models were created for the overall sample and stratified by sex. These models tested the influence of three anthropometric measures on MVPA (z-score waist circumference (zWC), z-score waist-to-height ratio (zWHR), and z-score Body Mass Index (zBMI)), with each anthropometric measure being tested in its own model (Model 1—zWC; 2—zWHR; and 3—zBMI). Each anthropometric measure was modeled separately rather than simultaneously. The three measures are derived from overlapping anthropometric inputs and are strongly correlated; entering them together would introduce substantial collinearity and yield coefficients that do not correspond to any measure used in practice, since screening programs use one measure at a time. Each model therefore reflects the information available with a single measure, and all models share the same covariate set (age in months, sex where applicable, accelerometer wear time, and school). Because the models are non-nested and share the same outcome and covariates, they are compared descriptively by the magnitude of the exposure coefficients, the proportion of variance explained, and the root mean square of the error, rather than by formal inferential testing. All three exposures are expressed as age- and sex-standardized z-scores, so their coefficients are on a common scale and represent the change in the outcome associated with a one-standard-deviation increase in each measure.

Linear regression models were also used to assess the association between anthropometric z-scores and time spent in sedentary, light, moderate, and vigorous physical activity intensities, in minutes per day. Logistic regression was used to model the probability of participants meeting the recommended MVPA threshold of 60 min per day. Total MVPA was chosen a priori as the primary outcome. Models of intensity-specific physical activity and of the probability of meeting the MVPA guideline were considered secondary, and the sex-stratified models exploratory. No formal correction for multiple comparisons was applied, as the analyses were intended to characterize a consistent pattern of association across related outcomes rather than to test a family of independent hypotheses. Models were stratified by sex a priori. Formal interaction terms between sex and each anthropometric measure were not fitted, and the stratified estimates should therefore be interpreted as describing associations within each sex rather than as establishing that the associations differ between sexes. The sex-stratified analyses are exploratory. All models included statistical adjustments for potential confounding variables: sex, wear time, age, and school. Because participants were recruited from four schools, multilevel models with a random school intercept were considered. With only four clusters, however, the between-school variance component cannot be estimated with acceptable precision, and random-intercept models with few clusters are known to produce unstable variance estimates and anti-conservative standard errors; cluster-robust standard errors are subject to the same limitation at this number of clusters. School was therefore included as a fixed effect in all models, which absorbs between-school variation without requiring estimation of a variance component.

Heteroscedasticity-consistent standard errors were used for all linear models, so inference does not rely on an assumption of constant error variance. Residual diagnostics showed evidence of non-normality when moderate or vigorous physical activity was the outcome based on the Shapiro–Wilk test. This is expected given zero inflation for inactive children and right skewness for highly active children. However, given the sample size of 264 individuals and the use of heteroscedasticity-consistent standard errors, our parameter estimates and inferences remain robust. There were also no major concerns with influential observations: the largest Cook’s distance was 0.103, well below standard thresholds of concern. All statistical analyses were conducted using the R (v. 4.4.1) environment for statistical computing [48]. The significance level was set at 0.05.

## 3. Results

Characteristics of the sample (*N* = 264) stratified by sex (55% girls) are displayed in Table 1. Table 2 indicates that zWC was significantly and negatively associated with total MVPA (*p* < 0.05). Age was a significant negative predictor of MVPA (*p* < 0.001). Being a boy was positively associated with MVPA (*p* < 0.001). None of the anthropometric z-scores were associated with MVPA among boys. The only significant variable in the sample of boys was age, being a negative predictor of MVPA (*p* < 0.001). Among girls, all anthropometric measures were significantly and negatively associated with MVPA. Older age and attending school 3 and 4 were significantly associated with lower MVPA in girls. Across models, zWC demonstrated larger effect sizes and higher R^2^ values for predicting MVPA and vigorous physical activity than zWHR and zBMI.

Table 3 shows no statistically significant associations between any anthropometric z-score and time spent in sedentary or light physical activity. zWC and zWHR showed the largest negative associations with vigorous physical activity. In boys, both zWC (*p* < 0.05) and zWHR (*p* < 0.05) were significantly and inversely associated with vigorous physical activity. In girls, all three anthropometric measures showed a significant negative association with vigorous activity: zWC (*p* < 0.05), zWHR (*p* < 0.01), and zBMI (*p* < 0.05). R^2^ values for vigorous activity were the lowest, ranging from 0.074 (Boys) to 0.187 (Girls), while being highest for sedentary and light activity (ranging from 0.392 to 0.434). For total MVPA among girls, the zWC model explained 32.7% of the variance, compared with 30.2% for zWHR and 30.1% for zBMI (RMSE 14.17, 14.43, and 14.44 min/day, respectively). For vigorous physical activity in the overall sample, the corresponding values were R^2^ = 0.176 for zWC, 0.166 for zWHR, and 0.147 for zBMI. The ordering was consistent across outcomes, with zWC showing the largest coefficients and the highest R^2^ values, followed by zWHR and then zBMI. The differences between models were nonetheless small, amounting to roughly one to three percentage points of explained variance.

Overall, 18.9% of the schoolchildren met the daily MVPA recommendations (Table 4). In total, 30.5% (*N* = 36) of boys met the MVPA standard, compared to 9.6% (*N* = 14) of girls. The highest MVPA adherence was 28% in 5-year-olds, and the lowest was in 9-year-olds (8.7%).

As shown in Table 5, a higher zWC was significantly associated with a 3.2% decrease in the probability of meeting the MVPA guidelines (*p* < 0.05) in the whole sample. In all models, increasing age was associated with a reduced likelihood of meeting the guidelines in the overall sample (*p* < 0.01). In the models specific to boys, none of the anthropometric z-scores showed a statistically significant association with meeting the MVPA guidelines. In girls, all anthropometric models were significantly associated with a decreased probability of meeting the recommended MVPA guidelines. Increasing age was significantly associated with a decreased probability of meeting the MVPA guidelines in the girl sample.

## 4. Discussion

The current work provides novel data on the associations of device-measured physical activity in predominantly African American schoolchildren living in high-risk neighborhoods. Our principal finding was that central adiposity, measured by zWC and zWHR, was significantly and negatively associated with MVPA and vigorous physical activity, as well as the likelihood of meeting recommended MVPA guidelines, among girls but not among boys. Because formal interaction terms were not tested, this pattern indicates where the associations reached statistical significance rather than a confirmed difference between sexes. The current data, along with critically low overall adherence to the MVPA guidelines, highlight a significant public health concern.

The decision to utilize WC and WHR was motivated by the limitations of BMI [49,50], which can underestimate overall body fat in children [8,9]. Furthermore, central body fat (as measured by WC and WHR) has been consistently shown to predict cardiovascular disease risk factors in children [51], whereas a strict BMI cutoff may not capture this. In our data, zWC and zWHR showed inverse associations with vigorous physical activity and with the likelihood of meeting MVPA guidelines that were of similar or slightly greater magnitude than those for zBMI, particularly among girls, though the difference between measures was small. Workshops led by the National Collaborative on Childhood Obesity Research have emphasized the need for obesity-related measures that are feasible, scalable, and meaningful across surveillance, epidemiologic, and intervention contexts [52]. Our findings support the use of waist measures as such tools, particularly in school-based settings and high-risk populations. Given that increased visceral adiposity markers (such as zWHR and zWC) are associated with adverse health outcomes [53], the significant negative associations observed herein reinforce the usefulness of these relatively simple measures in routine health screenings. In support of this data, earlier work has shown that waist circumference was deemed “most important” in mitigating the harmful effects of obesity in overweight Brazilian schoolchildren [54]. Future interventions targeting this population may consider using zWC and zWHR to identify children at elevated risk for physical inactivity and to evaluate whether physical activity programming mitigates adiposity-related differences in physical activity.

The differences in model performance between waist-based measures and BMI, while consistent in direction, were modest. At the level of an individual child, a difference of one to three percentage points in explained variance would be unlikely to alter how that child is characterized, and it would not justify replacing BMI with a waist-based measure on the strength of these data alone. The potential relevance of such a difference lies at the population and surveillance level, where a measure more consistently associated with the outcome of interest may identify a partially different group of children for follow-up across a large screening program. We therefore frame waist-based measures as a feasible complement to BMI in school-based surveillance rather than a substitute, and we note that whether their use would improve the targeting or effectiveness of physical activity interventions is an empirical question that this study was not designed to answer.

Among girls, all three anthropometric measures were significantly and inversely associated with total MVPA and the probability of meeting the 60-min guideline, whereas no anthropometric measure was significant among boys. Other studies examining weight status and waist circumference in relation to MVPA in boys have similarly not detected significant associations [55,56]. This contrast should be interpreted cautiously. The estimates themselves were not markedly different between sexes, for zWC and total MVPA, −3.334 (95% CI −5.49 to −1.18) among girls compared with −2.352 (95% CI −6.23 to 1.53) among boys. The smaller boy sample yielded less precise estimates, and the difference we observe is therefore one of statistical significance within strata rather than a demonstrated differential association. We report the sex-stratified findings because the pattern is consistent across outcomes and because sex differences in fat patterning and activity behavior are well documented, but we do not claim that adiposity is more strongly associated with physical activity in girls than in boys, and formal testing in a larger sample would be required to address that question. One potential explanation is that female anatomy favors more subcutaneous fat and less visceral fat, with a preference for storage in the hip area. At the same time, males store more visceral fat in the belly/waist region [57]. Among girls, higher central adiposity was associated with lower MVPA. The design does not allow us to determine the direction of this relationship, and lower physical activity contributing to the accumulation of central adiposity is at least as plausible an explanation as the reverse.

However, not all studies show a null effect of WC and WHR on MVPA in children overall [58]. The current work had many similarities with the Martinez-Gomez et al. study, which used actigraphy for seven days [58]. However, their study was conducted with ~97% Caucasian children, a different population from the current work. This may point to a racial/ethnic difference, potentially due to how body fat is stored, as research suggests white children may store more visceral fat than African American children [59,60].

When examining specific physical activity intensities (Table 3), significant associations were mainly observed with vigorous physical activity. This confirms existing evidence that vigorous physical activity tracks more closely with adiposity markers than less intense activity [37,46,61,62,63]. Notably, no significant associations were found between any anthropometric z-score and sedentary or light physical activity, which differs from research suggesting that reducing sedentary behavior and increasing light physical activity are primary steps in managing pediatric obesity [28,64,65]. Our findings indicate that vigorous physical activity was the intensity most strongly associated with central adiposity in this age group. Whether prioritizing vigorous activity within an intervention would alter adiposity cannot be inferred from these data and warrants prospective evaluation.

The overall adherence to the standard recommendation of 60 min of MVPA per day was 18.9%. This is similar to what is reported in low-income Brazilian children who are overweight or have obesity [44]. However, this is lower than the commonly reported 25% found among children [66,67]. Unfortunately, this highlights prevalent physical inactivity, even in a state with a 40-min/day recess minimum (Arkansas law (Act 641)). Adherence was markedly stratified by both sex and age, with boys roughly three times as likely as girls to meet the guideline and adherence declining steadily across the age range (Table 1 and Table 4). These results align with previous findings reporting age-related declines in PA [68,69] and demonstrate that the general trend of low PA and sex gaps persists, and is possibly amplified, within this low-income, African American cohort. Hence, there is a need for sex-specific interventions targeting older elementary school-aged girls.

The primary strengths of this work lie in the device-based measurement of free-living physical activity using accelerometry over an extended period. It focuses on a community-based sample of predominantly minority, low-income schoolchildren across four different schools, in neighborhoods at high risk for cardiometabolic outcomes, and may benefit from data-informed physical activity interventions. This study is cross-sectional; therefore, we cannot infer causality or rule out that lower activity levels may also contribute to adiposity accumulation over time. Additionally, the models, particularly for boys’ total MVPA, had low R^2^ values (12.4% to 13.3%), indicating that the measured anthropometric and demographic factors explain a minor fraction of the variance in physical activity. This suggests that the primary determinants of MVPA in this cohort are likely complex and that unmeasured confounders are present. For example, we observed a significant association between attending school 3 (for girls) and school 4 (for the overall sample and girls) and lower MVPA, suggesting the influence of environmental or institutional school-level factors that warrant further investigation. Potential areas for future investigation that were not included comprise: pubertal status, biological maturity, and maturational timing influence both fat patterning and physical activity behavior independently of chronological age. Dietary intake and screen time were not captured either, each of which may independently affect physical activity and covary with adiposity.

Waist circumference was measured at the umbilicus, consistent with the protocol applied in a prior study of German schoolchildren [34]. Other research investigations measure at the iliac crest. These sites are strongly correlated, but not interchangeable. Hence, direct comparison of our absolute waist values with studies using other landmarks is limited.

Sleep diaries were returned by 36% of parents. Rest intervals for the remaining participants were identified by two independent coders through visual inspection of accelerometer traces, an approach validated against diary-based classification in hip-worn accelerometer data collected under 24-h protocols, with a third coder adjudicating intervals that differed by more than 15 min between coders. Non-wear time was identified separately using the Troiano algorithm, and wear time was adjusted for in all models. Any residual misclassification of sleep and wake intervals is therefore likely to be modest and non-differential with respect to adiposity, which would bias associations toward the null rather than away from it. We cannot exclude the possibility that diary completion was itself associated with unmeasured household characteristics that also relate to physical activity.

Lastly, because no correction for multiple comparisons was applied, the probability that at least one nominally significant result reflects type I error is not negligible, and individual *p* values close to the 0.05 threshold should be interpreted with corresponding caution.

In this cross-sectional sample of predominantly African American, low-income schoolchildren, greater central adiposity was associated with lower device-measured physical activity and a lower probability of meeting physical activity guidelines; these associations were most consistent for vigorous activity and reached statistical significance among girls but not among boys. Waist-based z-scores showed associations with physical activity of similar or slightly greater magnitude than BMI, though each measure was modeled separately and the differences in model fit were modest, so these results do not establish that one measure is superior to another. The direction of the observed associations cannot be determined from this design, and lower physical activity contributing to the accumulation of central adiposity remains an equally plausible explanation. Set against these limitations, the descriptive finding is nonetheless striking: fewer than one in five children met the recommended 60 min of daily MVPA, and fewer than one in ten girls did so. These findings support further evaluation of waist-based measures as a low-cost complement to BMI in school-based surveillance and indicate a need for prospective studies to establish the direction of the adiposity–activity relationship in this population.

## Figures and Tables

**Table 1:** Characteristics of all children in the sample (*N* = 264) and stratified by sex.

	All		Boys		Girls	
Measure	Mean or *N*	SD or %	Mean or *N*	SD or %	Mean or *N*	SD or %
Total MVPA (min/d)	43.8	22.52	50.71	26.09	38.23	17.33
Sedentary (min/d)	378.45	88.36	377.61	85.2	379.12	91.13
Light (min/d)	450.8	76.8	446.39	69.56	454.36	82.25
Moderate (min/d)	34.58	16.32	39.17	18.18	30.88	13.62
Vigorous (min/d)	9.22	8.08	11.54	10.2	7.35	5.16
zWC	0.59	1.1	0.49	1.03	0.67	1.15
zWHR	0.37	1.12	0.33	1.07	0.4	1.16
zBMI	0.86	1.15	0.72	1.19	0.98	1.11
Wear time (min/d)	873.05	64.46	874.71	58.74	871.7	68.9
Age (months)	102.2	21.28	103.33	19.89	101.29	22.36
Sex (*N*, %)						
Female	146	55.3	0	0	146	100
Male	118	44.7	118	100	0	0
School						
1	61	23.1	30	25.4	31	21.2
2	68	25.8	32	27.1	36	24.7
3	74	28	29	24.6	45	30.8
4	61	23.1	27	22.9	34	23.3

MVPA, moderate–vigorous physical activity; zWC, z-score waist circumference; zWHR, z-score waist-to-height ratio; zBMI, z-score body mass index; SD, standard deviation; *N*, number of participants; min/d, minutes per day.

**Table 2:** Regression analysis of factors associated with total moderate–vigorous physical activity (MVPA).

	All			Boys			Girls		
	(1)	(2)	(3)	(1)	(2)	(3)	(1)	(2)	(3)
Intercept	60.175 *** (15.083)	60.966 *** (15.300)	63.966 *** (15.347)	92.618 ** (32.986)	92.388 ** (33.336)	94.593 ** (32.872)	47.011 ** (13.470)	49.253 ** (13.827)	52.570 ** (14.563)

zWC	−2.813 * (1.023)			−2.352 (1.969)			−3.334 ** (1.095)		
						
zWHR		−2.012 (0.966)			−1.843 (1.829)			−2.257 * (1.036)	
						
zBMI			−1.190 (0.987)			−0.140 (1.683)			−2.265 * (1.124)
						
Wear time (min/d)	0.025 (0.017)	0.023 (0.017)	0.021 (0.017)	0.003 (0.039)	0.001 (0.039)	−0.000 (0.039)	0.039 * (0.015)	0.036 (0.015)	0.036 (0.016)

Sex (male)	12.478 *** (2.627)	12.847 *** (2.609)	12.687 *** (2.689)						
						
Age (months)	−0.370 *** (0.058)	−0.371 *** (0.058)	−0.385 *** (0.058)	−0.391 ** (0.121)	−0.383 ** (0.122)	−0.396 ** (0.122)	−0.346 *** (0.056)	−0.355 *** (0.056)	−0.370 *** (0.057)

School 2	−3.479 (3.908)	−3.518 (3.948)	−3.177 (3.986)	−2.684 (7.011)	−2.846 (7.030)	−2.356 (7.109)	−4.065 (3.819)	−4.016 (3.933)	−4.035 (4.008)

School 3	−3.862 (3.952)	−3.943 (3.972)	−3.990 (4.028)	1.494 (7.366)	1.411 (7.370)	1.241 (7.425)	−7.777 * (3.933)	−7.863 * (4.011)	−7.940 * (4.044)

School 4	−9.013 * (3.700)	−9.090 * (3.720)	−9.200 * (3.736)	−9.455 (6.547)	−9.419 (6.563)	−9.215 (6.586)	−9.002 * (3.956)	−9.255 * (4.017)	−9.668 ** (4.023)

*N*	264	264	264	118	118	118	146	146	146
R^2^	0.251	0.242	0.236	0.133	0.130	0.124	0.327	0.302	0.301
RMSE	19.45	19.56	19.64	24.20	24.24	24.31	14.17	14.43	14.44

MVPA, moderate–vigorous physical activity; zWC, z-score waist circumference; zWHR, z-score waist-to-height ratio; zBMI, z-score body mass index; *N*, number of observations; RMSE, root mean square error; R^2^, coefficient of determination; SE, standard error. Parentheses indicate heteroskedasticity-robust standard errors. * *p* < 0.05, ** *p* < 0.01, *** *p* < 0.001.

**Table 3:** Regression analysis of the association between anthropometrics and time spent (in minutes) in physical activity intensities.

	**All (*N* = 264)**											
	**Sedentary**			**Light**			**Moderate**			**Vigorous**		
	**(1)**	**(2)**	**(3)**	**(1)**	**(2)**	**(3)**	**(1)**	**(2)**	**(3)**	**(1)**	**(2)**	**(3)**
zWC	3.730 (3.946)			−0.917 (3.522)			−1.326 (0.774)			−1.487 *** (0.344)		
								
zWHR		1.337 (3.675)			0.675 (3.341)			−0.742 (0.733)			−1.270 ** (0.331)	
								
zBMI			−1.525 (3.527)			2.715 (3.111)			−0.466 (0.754)			−0.724 (0.350)
								
R^2^	0.415	0.413	0.413	0.392	0.392	0.394	0.264	0.259	0.257	0.176	0.166	0.147
	**Boys (*N* = 118)**											
	**Sedentary**			**Light**			**Moderate**			**Vigorous**		
zWC	−0.293 (6.319)			2.644 (5.714)			−0.179 (1.464)			−2.173 * (0.693)		
								
zWHR		1.021 (5.744)			0.822 (5.268)			−0.037 (1.394)			−1.806 * (0.630)	
								
zBMI			−3.115 (5.166)			3.256 (4.478)			0.592 (1.283)			−0.732 (0.641)
								
R^2^	0.407	0.407	0.409	0.328	0.326	0.329	0.161	0.161	0.162	0.114	0.102	0.074
	**Girls (*N* = 146)**											
	**Sedentary**			**Light**			**Moderate**			**Vigorous**		
zWC	6.329 (5.262)			−2.995 (4.671)			−2.204 ** (0.824)			−1.131 ** (0.370)		
								
zWHR		1.420 (4.894)			0.837 (4.399)			−1.307 (0.764)			−0.950 ** (0.371)	
								
zBMI			0.018 (4.905)			2.247 (4.414)			−1.500 (0.866)			−0.766 * (0.360)
								
R^2^	0.425	0.420	0.419	0.434	0.432	0.433	0.339	0.318	0.321	0.187	0.170	0.154

zWC, z-score waist circumference; zWHR, z-score waist-to-height ratio; zBMI, z-score body mass index; R^2^, coefficient of determination; SE, standard error; min/d, minutes per day. All models included adjustments for wear time, school, age in months, and, if applicable, sex. Numbers in parentheses depict robust standard errors. * *p* < 0.05, ** *p* < 0.01, *** *p* < 0.001.

**Table 4:** Met recommended 60 min per day of MVPA.

	No	Yes	% Meeting
Age (years)			
5	18	7	28
6	28	10	26.3
7	24	9	27.3
8	41	13	24.1
9	42	4	8.7
10	43	5	10.4
11–12	18	2	10
Sex			
Female	132	14	9.6
Male	82	36	30.5
Overall	214	50	18.9

MVPA, moderate–vigorous physical activity.

**Table 5:** Marginal effects of anthropometric exposures on the probability of meeting the 60-min MVPA guideline.

	All (*N* = 264)			Boys (*N* = 118)			Girls (*N* = 146)		
	(1)	(2)	(3)	(4)	(5)	(6)	(7)	(8)	(9)
zWC	−0.032 * (0.014)			−0.005 (0.039)			−0.088 *** (0.024)		
						
zWHR		−0.027 (0.014)			0.000 (0.038)			−0.079 ** (0.024)	
						
zBMI			−0.016 (0.012)			0.011 (0.034)			−0.069 ** (0.021)
						
Wear time (min/day)	0.000 (0.000)	0.000 (0.000)	0.000 (0.000)	0.001 (0.001)	0.001 (0.001)	0.001 (0.001)	0.001 (0.000)	0.001 (0.000)	0.001 (0.000)

Sex (male)	0.209 *** (0.046)	0.215 *** (0.047)	0.210 *** (0.047)						
						
Age (months)	−0.002 ** (0.001)	−0.002 ** (0.001)	−0.003 ** (0.001)	−0.005 * (0.002)	−0.005 * (0.002)	−0.005 * (0.002)	−0.003 ** (0.001)	−0.003 * (0.001)	−0.004 ** (0.001)

School 2	0.006 (0.042)	0.005 (0.043)	0.011 (0.043)	0.127 (0.116)	0.128 (0.116)	0.131 (0.116)	−0.100 (0.067)	−0.101 (0.071)	−0.112 (0.072)

School 3	−0.025 (0.040)	−0.025 (0.040)	−0.026 (0.040)	0.068 (0.116)	0.068 (0.116)	0.067 (0.117)	−0.133 * (0.063)	−0.132 * (0.067)	−0.158 * (0.066)

School 4	−0.033 (0.041)	−0.032 (0.042)	−0.033 (0.042)	−0.057 (0.115)	−0.056 (0.115)	−0.055 (0.116)	−0.026 (0.083)	−0.029 (0.087)	−0.042 (0.088)


MVPA, moderate–vigorous physical activity; zWC, z-score waist circumference; zWHR, z-score waist-to-height ratio; zBMI, z-score body mass index. * *p* < 0.05, ** *p* < 0.01, *** *p* < 0.001.

## Data Availability

This article is part of a broader and ongoing four-year study. The data presented in this study are confidential during the study period; deidentified data will be made available in a public repository at the end of the study period.
